# Association Between Quality of Life and Drug Adherence Among Patients With Diabetes in India

**DOI:** 10.7759/cureus.71300

**Published:** 2024-10-12

**Authors:** Hitesh Chaudhari, Barna Ganguly, Nazima Mirza

**Affiliations:** 1 Pharmacology, Pramukhswami Medical College, Anand, IND

**Keywords:** antidiabetic medications, diabetes mellitus(dm), quality of life (qol), whoqol-bref, medication adherence

## Abstract

Introduction: Diabetes represents a significant global health concern. Effective diabetes management necessitates substantial behavioral and psychological modifications, including regular glucose monitoring, dietary adjustments, and medication adherence. However, treatment adherence remains suboptimal, particularly in developing countries, leading to poor glycaemic control, increased healthcare costs, and diminished quality of life (QoL). Common factors influencing medication nonadherence include lack of medication knowledge, age, education of patients, polypharmacy, duration of disease, cost of therapy, complexity of dosing regime, and the presence of comorbidities. So, the study aims to assess the adherence status to antidiabetic medication and its association with QoL among diabetes patients in the Anand district of Gujarat state.

Methodology: A study was carried out in Anand district, Gujarat, from 2023 to 2024, focusing on community-based cross-sectional research. In the eight blocks of Anand district, a two-stage cluster sampling method was utilized, with one village or town randomly chosen from each taluka, and 25 diabetes patients interviewed per area. Data collection involved visiting 200 participants' houses, starting with a randomly selected house and continuing until the target was met. The interviews, conducted in the local language, lasted around 45 minutes and utilized a pre-validated and pretested questionnaire. The questionnaire covered demographic details, disease characteristics, and medication adherence using the semi-structured scale from Adherence to Refills and Medications Scale (ARMS-D), as well as the assessment of QoL using the WHOQoL-BREF questionnaire. Descriptive and inferential statistics were used for data analysis. Sociodemographic attributes were presented in terms of percentages (%) and frequencies (N). The means and standard deviation (SD) of health-related QoL and adherence to anti-diabetic medication were reported. Multiple linear regression analysis was used to assess the impact of adherence to antidiabetic medication within each QoL area after adjusting for a few sociodemographic variables. P values below 0.05 were considered statistically significant.

Results: The study included predominantly older participants (58.1 years) with 53.5% being male. Most participants (65.5%) had diabetes for more than 5 years, 60.0% had comorbidities, and 29.0% experienced complications. Nonadherence to treatment was observed in 37.0% of patients. The study found that factors like age, education, occupation, family history of diabetes, smoking, alcohol consumption, exercise habits, and medication adherence significantly influenced the QoL in diabetic patients, with medication adherence showing the strongest positive association with all QoL domains.

Conclusion: This study shows a high percentage of nonadherence to antidiabetic medications. Poor adherence had significantly lower mean scores across all domains of QoL. Community-based educational programs targeting older and less-educated individuals should promote regular physical activity. Developing strategies to improve medication adherence and ensuring affordable access to essential medications, along with prioritizing enhancements in the QoL through psychosocial support and lifestyle counseling are imperative for diabetic patients.

## Introduction

Diabetes, an increasingly common metabolic condition characterized by elevated glucose levels, presents a significant health issue due to its growing prevalence. According to the International Diabetes Federation (IDF), the global diabetes prevalence was reported to be 10.5% in 2021, affecting approximately 536.6 million people. It is estimated that diabetes will lead to about 6.7 million deaths in the 20-79 age group in 2021 [1]. The percentage of people with diabetes in India is projected to increase from 9.6% in 2021 to 10.4% by 2030 [1].

Managing diabetes requires significant behavioral and psychological adjustments, including regular blood sugar monitoring, dietary changes, and medication adherence [2]. Treating chronic illnesses requires long-term treatment if failed, increasing the risk of long-term consequences. Developing nations face challenges in providing continuous diabetes care due to the emphasis on treating infectious diseases [3].

Quality of life (QoL) is a key measure for evaluating management plans for any illness. Effective diabetes control involves drug therapy and lifestyle adjustments, with medication compliance essential for desired clinical outcomes. Treatment nonadherence impedes effective diabetes care, resulting in suboptimal glycemic control [4]. Inadequate treatment causes a lower QoL for patients, increased reliance on acute care and hospitalization, as well as higher expenditures on medications and medical services [5].

In 2003, the World Health Organization (WHO) emphasized that "improving the effectiveness of adherence interventions may have a far greater impact on the population's health than any improvement in specific medical treatments" [6]. Medication non-adherence stands as a significant public health issue and is notably widespread among individuals with diabetes [7]. Noncompliance with antidiabetic treatment is often linked to poor metabolic control, resulting in short-term and long-term consequences [6,8]. Patients' nonadherence to anti-diabetic medication compromises its safety and efficacy, elevating the likelihood of illness and death leading to increased healthcare costs and decreasing the overall QoL. A previous study on diabetes found a link between patients' QoL and their medication adherence. It suggested that adhering to treatment regimens may improve the QoL for individuals with chronic conditions, while non-adherence may have the opposite effect [9]. Failure to follow medication schedules can have negative effects on QoL and increase the chances of complications and mortality.

The impact of diverse non-adherence rates to anti-diabetic medication on treatment outcomes is significant. Many individuals with diabetes do not follow the prescribed dosage regimen. Improving adherence status in diabetes treatment is a crucial public health concern. Medication non-adherence is more common in India due to limited access to medical facilities and low literacy rates [10]. Common factors influencing medication nonadherence include lack of medication knowledge, age, education of patients, polypharmacy, duration of disease, cost of therapy, complexity of dosing regime, and the presence of comorbidities [8].

The majority of research concerning medication nonadherence among diabetics in India comes from hospital studies. These studies indicate that nonadherence is common and is affected by behavioral and sociocultural factors [8]. Currently, there is insufficient research on how common it is for diabetes patients in the study area to not adhere to their medication, and how this non-adherence affects their QoL. There is a dearth of research data on the assessment of QoL and nonadherence to treatment in diabetic patients. To develop solutions to overcome the barriers, it is necessary to acquire relevant data on many different factors linked to medication nonadherence. The current research in the local area sought to establish how common it is for diabetic patients in the Anand district of Gujarat, India to not follow their prescribed antidiabetic medication and how their medication adherence status affects their QoL.

## Materials and methods

Study design and setting

A cross-sectional study was performed in the Anand district of Gujarat, India. It took place for one year, from March 2023 to February 2024. Ethics approval (IEC/BU/143/Faculty/12/70/2023; dated February 16, 2023) was obtained from the Institutional Ethics Committee-2, Pramukhswami Medical College, Anand.

Study population and sample size

This study included patients over 18 years old age with confirmed diabetes mellitus type-1 and type-2 for at least six months. A sample size of 200 was based on a previous study [11] estimated through cluster sampling, who did not take their anti-diabetic medications as prescribed with a level of significance = 5%, confidence level = 95%, acceptable difference = 10%, assumed Proportion = 0.454, design effect = 2, no. of clusters = 8.

Sampling technique

The study's sample was chosen using a two-stage cluster sampling technique. Each of all eight blocks (tehsil) of Anand district was considered a cluster. So, in the first stage, 8 clusters were made. In the second stage, any area (village/ town) from each cluster was selected randomly from each taluka. With the use of a consecutive sampling strategy, a total of 25 diabetes mellitus (Both type-1 and type-2) patients were recruited and interviewed from each selected area of the village/town as per inclusion and exclusion criteria. Thus, data from 200 diabetic mellitus patients were included in the study.

Inclusion and exclusion criteria

The study included patients, regardless of gender, who were at least eighteen years old and had been confirmed with diabetes mellitus type-1 and type-2 for at least six months following the first diagnosis. In addition, the patients had to meet the study's inclusion requirements, which included having a valid medical record confirming their diagnosis of diabetes and a prescription for anti-diabetic medications, as well as taking at least one medicine to treat their condition. Patients in critical condition, those whose physical or mental health could make it difficult for them to understand or complete questionnaires, and those whose main method of controlling their diabetes was diet rather than using anti-diabetic drugs on prescription were not allowed to participate in the study.

Sampling procedure

Data was collected by house-to-house visits from each selected area over one year. To establish contact, the chief of the villages and other known members of the communities were first visited before the start of the study. A study was started from any one house randomly in the selected area with a participant having diabetes and it was continued till the desired sample (25 diabetes patients) was achieved.

A person fitting in inclusion criteria was interviewed by an investigator after obtaining institutional ethics committee approval and written informed consent from the study participants. A brief detail regarding the study was discussed with each selected participant providing participants information sheet before the initiation of the interview. Each participant provided written informed consent, and their privacy and confidentiality were safeguarded. If there was more than one diabetic patient in a single household, one patient was chosen through a lottery method. The interview was held in the local language as per the specified questions. The pre-specified questions were prepared and validated (external as well as internal validation) before starting the study. The average time for conducting interviews was 45 minutes. During the interview, the participants were asked to bring and show the medicines available for diabetes and other diseases. Demographic, lifestyle, and disease characteristics were asked and then medicine-related details were asked and observations from the same were recorded in a case record form.

Data collecting tools

Data was collected by conducting a structured survey in both Gujarati and English languages. The survey is divided into three sections: demographics, lifestyle and illness characteristics, a scale for medication adherence, and the WHO Quality of Life (QoL) BREF scale.

The demographics, lifestyle, and disease characteristics section includes inquiries about gender, age, BMI, education, employment, marital status, smoking, alcohol consumption, type of diabetes, duration of diabetes, diabetes-related complications, presence of other health conditions, and the number of current anti-diabetic medications. The researchers utilized the Adherence to Refills & Medications Scale (ARMS-D), a semi-structured tool they developed, to assess non-adherence to anti-diabetic medication over the past two weeks [12]. The levels of adherence were evaluated using a scale for adherence to antidiabetic drugs [12]. The internal consistency of the scale was high, as indicated by a Cronbach’s alpha value of 0.91 for all 10 items. The total score of the scale falls within the range of 0 to 40, with nonadherence categorized as 31-40, mild adherence as 21-30, moderate adherence as 11-20, and high adherence as 0-10 (see Appendix A). Participant scores were noted.

We employed the WHOQoL-BREF from the World Health Organization to evaluate the QoL in individuals with diabetes. The WHOQoL-BREF is a validated instrument utilized across diverse population groups and cultural settings. This general tool is specifically developed to assess QoL and it is a shorter adaptation of the WHOQoL-100 [13]. The WHOQoL-BREF, a 26-item assessment tool developed by the World Health Organization, assesses the QoL across four domains: physical health, psychological, social relationships, and environment (see Appendix B). Most of the items utilize a 1 to 5 scale, with higher ratings indicating better QoL [14]. We reversed the negatively phrased items 3, 4, and 26 before conducting additional analysis. A high QoL is indicated by a high score, while a low QoL is indicated by a low scale. Following WHOQoL guidelines, the raw score for these four domains was converted to a 0-100 scale. Each item's score was multiplied by four to allow for direct comparison to WHOQoL-100.

To check translation accuracy, question flow, and interview feasibility, we conducted a pilot study with 10 patients who were not included in our main study. We made minor questionnaire changes, primarily related to formatting errors.

Statistical analysis

Data was transformed into a digital format and inputted into Microsoft Excel 365 (Microsoft Corp., Redmond, WA) spreadsheets. The software IBM SPSS Statistics for Windows, version 20 (IBM Corp., Armonk, NY) was utilized for statistical analysis, including both inferential and descriptive statistics. Sociodemographic characteristics were represented as percentages (%) and frequencies (N). QoL and adherence status to anti-diabetic medication were expressed as averages and standard deviations (SD). The association of anti-diabetic medication adherence on each dimension of QoL was evaluated using multiple linear regression analysis, adjusting for specific sociodemographic factors. P value < 0.05 was considered statistically significant.

## Results

A total of 221 houses with one diabetic patient were visited. Out of them, 21 were excluded, due to no available valid medical records or anti-diabetic drug details (11), participants with only dietary control and not taking any medications (seven) and three participants refused to participate in the study. So, the data of the 200 study participants were analyzed.

Table 1 shows the mean score of the physical, psychological, social relationship, environmental, overall perception of QoL, and overall perception of health. The highest mean score found in the psychological domain was 44.07 ± 22.61. The lowest mean score in the overall perception of QoL was 42.00 ± 26.38.

**Table 1:** QoL of study participants. Data are presented as mean ± SD (domain scores). QoL: quality of life.

QoL domains	Mean ± SD
Physical domain	43.94 ± 22.23
Psychological domain	44.07 ± 22.61
Social relationship domain	43.29 ± 24.67
Environment domain	42.81 ± 23.79
The overall perception of QoL	42.00 ± 26.38
The overall perception of health	42.13 ± 27.63

Table 2 shows the demographics of 200 study participants. The average age of the participants with diabetes was 58.1 ± 12.9 years, and the majority (52.5%) were above 60. There were slightly more male participants (107, 53.5%) than female participants (93, 46.5%). 31.5% reported being illiterate, 44.0% had completed education up to the secondary level and 20.5% were graduates. 39.0% were engaged in household work, 28.0% were unemployed, and 11.0%, each, were involved in office work, physical work, and retirement. Additionally, 79.5% were married, 14.5% were widows or widowers, and 5.0% were unmarried. Among the participants, 29.5% had a tobacco addiction, while 2.0% were alcoholics.

**Table 2:** QoL domain score according to sociodemographic characteristics of study participants. Data are presented as N (%) or mean ± SD, domain scores are mean ± SD. QoL: quality of life.

Characteristics	N (%)	Physical domain (Mean ± SD)	Psychological domain (Mean ± SD)	Social relationship domain (Mean ± SD)	Environment domain (Mean ± SD)	The overall perception of QoL (Mean ± SD)	The overall perception of health (Mean ± SD)
Age (year)							
18 to 30	07 (03.5)	75 ± 11.8	79.2 ± 12.5	72.6 ± 15.8	80.4 ± 14.1	71.4 ± 17.3	78.6 ± 17.3
31 to 60	88 (44.0)	52 ± 22.8	51.4 ± 22.5	50.4 ± 25.6	50.7 ± 23.7	50.6 ± 26.5	49.1 ± 28
> 60	105 (52.5)	35.1 ± 17.5	35.6 ± 18.6	35.4 ± 21	33.7 ± 19.3	32.9 ± 22.8	33.8 ± 24.3
Gender							
Male	107 (53.5)	44.1 ± 21.4	44.1 ± 22.9	44.4 ± 24.9	43.4 ± 23.5	41.8 ± 25.9	42.1 ± 26.1
Female	93 (46.5)	43.8 ± 23.2	44 ± 22.4	42 ± 24.4	42.1 ± 24.2	42.2 ± 27.1	42.2 ± 29.5
Education							
Illiterate	63 (31.5)	27.2 ± 9.7	26.7 ± 10.8	22.8 ± 11	24.6 ± 11	25 ± 14.9	23.4 ± 15.5
Up to secondary	88 (44.0)	42.7 ± 17.4	43 ± 17.3	44.4 ± 20.4	41.8 ± 18.6	41.5 ± 22.1	42.9 ± 22.7
Graduate	41 (20.5)	66.7 ± 21.9	67.1 ± 21.3	65.2 ± 21.4	66.3 ± 23.2	65.2 ± 29.5	64 ± 31.1
Postgraduate and above	08 (4.0)	72.8 ± 13.5	74 ± 18.2	80.2 ± 14.8	77.4 ± 9.1	62.5 ± 23.1	68.8 ± 29.1
Occupation							
Household work	78 (39.0)	39.5 ± 20.7	39.1 ± 19.6	36.1 ± 21.9	37 ± 22.1	37.5 ± 25.4	38.5 ± 28.4
Office work	22 (11.0)	72.4 ± 19.7	73.5 ± 19.5	72.4 ± 23.5	72.3 ± 21.9	67 ± 30.3	68.2 ± 28
Physical work	22 (11.0)	51.9 ± 12.8	52.8 ± 16.2	53 ± 19.7	53.3 ± 11.8	53.4 ± 14	46.6 ± 20.8
Retired	22 (11.0)	51.3 ± 21.1	54 ± 21.6	56.4 ± 24	53.7 ± 23.6	47.7 ± 28.8	45.5 ± 32.4
Unemployed	56 (28.0)	33 ± 16.8	32.1 ± 17.1	32.9 ± 18.1	30.9 ± 16.8	31.7 ± 20.5	33.9 ± 19.9
Marital status							
Unmarried	10 (05.0)	44.6 ± 27.9	44.2 ± 24.3	38.3 ± 32.7	44.7 ± 28.8	45 ± 32.9	37.5 ± 41.2
Married	159 (79.5)	46.2 ± 21.8	46.2 ± 22.7	46.4 ± 24.1	45.1 ± 23.7	44.2 ± 26.1	44.7 ± 27.2
Widow/widower	29 (14.5)	32.8 ± 19.8	34.3 ± 19.1	30.5 ± 19.7	31.7 ± 19.1	30.2 ± 23.5	31 ± 22.8
Divorced/separated	02 (01.0)	21.4 ± 0	16.7 ± 0	8.3 ± 0	12.5 ± 0	25 ± 0	25 ± 0
Any form of tobacco addiction							
Present	59 (29.5)	35.8 ± 17.3	36.1 ± 20.1	34.6 ± 20.6	35.3 ± 20.3	33.5 ± 22.6	32.6 ± 21.4
Absent	141 (70.5)	47.3 ± 23.2	47.4 ± 22.8	46.9 ± 25.4	46 ± 24.5	45.6 ± 27.1	46.1 ± 29
Alcohol addiction							
Yes	04 (02.0)	43.2 ± 21.8	43.3 ± 22.2	42.3 ± 23.9	42 ± 23.4	41.6 ± 26.3	41.3 ± 27.3
No	196 (98.0)	80.3 ± 3.6	80.2 ± 10.4	91.7 ± 0	82.1 ± 1.6	62.5 ± 25	81.3 ± 12.5

Table 2 displays the QoL domain scores based on the sociodemographic characteristics of participants. Younger participants (aged 18 to 30) consistently reported higher mean scores across all QoL domains compared to middle-aged and older participants. Mean scores for each QoL domain were similar between gender groups. Illiterate participants had the lowest mean scores, and as education level increased, mean scores for each domain of QoL also increased. Participants engaged in office work reported the highest mean scores in the physical domain, followed by those involved in physical work and retired individuals. Married participants reported the highest mean scores across all domains of QoL, and participants with addiction to tobacco or alcohol had lower mean scores in contrast to those with no addiction.

In Table 3, the BMI categories of the 200 study participants are shown. The majority (112, 56.0%) had a normal BMI, 86 (43.0%) were overweight, and one participant (0.5%) was obese and underweight, respectively. The average BMI was 24.36 ± 2.04 kg/m2. The table also shows the duration of exercise done by the participants. The majority (160, 80.0%) reported not doing any exercise, while smaller proportions engaged in various hours of exercise per week.

**Table 3:** QoL domain score with the status of BMI and duration of exercise of study participants. Data are presented as N (%) or mean ± SD, domain scores are mean ± SD. BMI: body mass index (kg/m2), QoL: quality of life.

Characteristics	N (%)	Physical domain (Mean ± SD)	Psychological domain (Mean ± SD)	Social relationship domain (Mean ± SD)	Environment domain (Mean ± SD)	The overall perception of QoL (Mean ± SD)	The overall perception of health (Mean ± SD)
BMI (kg/m^2^)							
Underweight	01 (0.5)	10.7 ± 0	12.5 ± 0	16.7 ± 0	9.4 ± 0	10 ± 0	25 ± 0
Normal	112 (56.0)	46.9 ± 22.8	47.6 ± 22.7	45.7 ± 23.9	45.6 ± 24.1	45.8 ± 25.5	46.2 ± 27.3
Overweight	86 (43.0)	40.4 ± 21	40 ± 21.8	40 ± 25.1	39.4 ± 23	37.5 ± 26.8	37.5 ± 27.3
Obese	01 (0.5)	46.4 ± 0	33.3 ± 0	83.3 ± 0	59.4 ± 0	50 ± 0	42 ± 0
Duration of exercise (hours/week)							
0	160 (80.0)	37.4 ± 18.5	37.2 ± 18.5	37 ± 22.3	35.8 ± 20	34.8 ± 22.3	35.3 ± 24.7
1 to 10	02 (1.0)	57.2 ± 15.2	58.4 ± 11.8	70.8 ± 17.7	64.1 ± 2.2	62.5 ± 17.7	50 ± 35.4
11 to 15	20 (10.0)	69.3 ± 18.1	69.4 ± 17.7	67.5 ± 18.5	68.3 ± 17.1	71.3 ± 23.3	67.5 ± 23.1
16 to 20	15 (7.5)	72.4 ± 14.3	74.7 ± 13.4	70.6 ± 14	74.2 ± 15.4	71.7 ± 20.8	73.3 ± 20
> 20	03 (1.5)	71.4 ± 12.4	79.2 ± 7.2	63.9 ± 9.6	77.1 ± 9	66.7 ± 28.9	75 ± 0

Table 3 shows QoL domain scores by BMI and exercise duration. Underweight individuals had the lowest scores. Normal-weight individuals generally reported moderate to high scores. Overweight individuals had scores similar to normal BMI, but slightly lower in Overall Perception of QoL and Health. Individuals who were obese had varying outcomes, exhibiting higher scores in the physical, social relationship, and environment domains, but lower scores in the psychological domain. Participants without exercise had lower scores across all domains compared to those with exercise. Individuals with moderate and high levels of exercise had higher scores across all QoL domains.

Table 4 summarizes the findings of a study with 200 participants. It shows that 10 (5.0%) have type 1 diabetes and 190 (95.0%) have type 2 diabetes. It shows that 69 individuals (34.5%) had diabetes for six months to five years, while 131 individuals (65.5%) had it for over five years. Among the participants, 89 (44.5%) had a family history of diabetes, while 111 (55.5%) did not; 58 (29.0%) had diabetes complications, while 142 (71.0%) did not. Among them, 120 (60.0%) had comorbidities, with hypertension being the most common.

**Table 4:** QoL domain score and diabetic profile of the study participants. Data are presented as N (%) or mean ± SD, domain scores are mean ± SD. QoL: quality of life.

Diabetic profile	N (%)	Physical domain (Mean ± SD)	Psychological domain (Mean ± SD)	Social relationship domain (Mean ± SD)	Environment domain (Mean ± SD)	The overall perception of QoL (Mean ± SD)	The overall perception of health (Mean ± SD)
Type of diabetes							
Type 1	10 (5.0)	81.8 ± 11	80.4 ± 11.8	81.7 ± 10.2	83.5 ± 12	80 ± 19.7	85 ± 17.5
Type 2	190 (95.0)	42 ± 20.9	42.2 ± 21.4	41.3 ± 23.5	40.7 ± 22.3	40 ± 25.2	39.9 ± 26.2
Duration of diabetes							
6 months to 5 years	69 (34.5)	52.7 ± 25.7	50.8 ± 27.4	50.5 ± 28.1	51 ± 28.2	48.9 ± 33.1	51.8 ± 30.7
> 5 years	131 (65.5)	39.3 ± 18.7	40.5 ± 18.8	39.5 ± 21.8	38.5 ± 19.9	38.4 ± 21.3	37 ± 24.5
Family history of diabetes							
Present	89 (44.5)	39.6 ± 20.9	39.8 ± 22.3	37.7 ± 25.8	37.6 ± 23.7	36.5 ± 25.9	37.6 ± 26.4
Absent	111 (55.5)	47.4 ± 22.7	47.5 ± 22.4	47.7 ± 22.8	47 ± 23.2	46.4 ± 26.1	45.7 ± 28.2
Complication							
Present	58 (29.0)	30.3 ± 17.1	29.3 ± 15.3	27.4 ± 20.1	28.1 ± 18.6	27.2 ± 20.9	25 ± 21.8
Absent	142 (71.0)	49.3 ± 21.8	49.8 ± 22.4	49.5 ± 23.5	48.5 ± 23.2	47.7 ± 26.1	48.8 ± 26.8
Comorbidity							
Present	120 (60.0)	34 ± 18	34.2 ± 18.5	33.3 ± 21.5	32.6 ± 20	33.8 ± 23.7	30.6 ± 23.9
Absent	80 (40.0)	58.9 ± 19.5	58.9 ± 20.1	58.2 ± 21.4	58.2 ± 20.6	54.4 ± 25.4	59.4 ± 23.7

In Table 4, participants with type 1 diabetes had higher mean scores in all QoL domains compared to those with type 2 diabetes. Those with a longer duration of diabetes (> 5 years) reported lower mean scores across all QoL domains, while participants with a family history of diabetes or complications had lower mean scores in QoL domains. Additionally, participants with comorbidities reported lower mean scores in all QoL domains.

Table 5 shows 74 (37.0%) study participants demonstrated nonadherence to treatment which falls in the 31 to 40 medication adherence score, while 56 (28.0%) had mild adherence (21-30 score), 42 participants (21.0%) had moderate adherence (11-20 score), and 28 participants (14.0%) had high adherence (01-10 score).

**Table 5:** QoL domain score with antidiabetic medication adherence score of study participants. Data are presented as N (%) or mean ± SD, domain scores are mean ± SD. QoL: quality of life.

Antidiabetic adherence score	N (%)	Physical domain (Mean ± SD)	Psychological domain (Mean ± SD)	Social relationship domain (Mean ± SD)	Environment domain (Mean ± SD)	The overall perception of QoL (Mean ± SD)	The overall perception of health (Mean ± SD)
31-40: nonadherence	74 (37.0)	22.59 ± 6.99	22.7 ± 7.33	19.04 ± 9.51	19.69 ± 8.31	20.27 ± 15.3	17.57 ± 13.55
21 -30: mild adherence	56 (28.0)	40.31 ± 7.14	39.73 ± 9.34	41.37 ± 11.46	39.02 ± 8.4	37.95 ± 13.48	39.29 ± 15.71
11-20: moderate adherence	42 (21.0)	60.88 ± 7.57	61.61 ± 6.42	63.9 ± 7.74	61.48 ± 5.2	58.93 ± 14.42	61.31 ± 16.75
1- 10: high adherence	28 (14.0)	82.26 ± 8.55	82.9 ± 7.11	80.35 ± 13.28	83.5 ± 6.95	82.14 ± 19.07	83.93 ± 12.2

**Figure 1:**
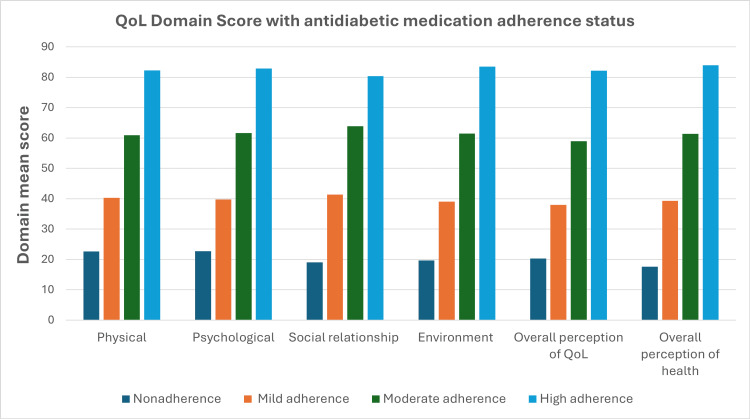
QoL domain mean score with antidiabetic medication adherence score. Data are presented as mean. QoL: quality of life.

Table 5 and Figure 1 depict the physical domain, mean scores increased significantly from nonadherence (22.59 ± 6.99) to high adherence (82.26 ± 8.55). Similarly, the mean of psychological, social relationship, and environmental domains scores also increased from nonadherence to high adherence. The overall perception of QoL mean score increased significantly from nonadherence (20.27 ± 15.3) to high adherence (82.14 ± 19.07) and also increased the overall perception of health mean score from nonadherence (17.57 ± 13.55) to high adherence (83.93 ± 12.2).

In Table 6, age was found to have a significant negative association with the physical, psychological, and environmental domains, as well as the overall perception of QoL, but not with the social relationship domain and overall perception of health. Gender did not show significant associations across any of the QoL domains. Higher education was positively associated with the social relationship domain, while other domains did not show significant associations. The current occupation was positively associated with the psychological, social relationship, and environment domains but had no significant association with the overall perception of QoL or health. Marital status did not significantly impact any of the QoL domains. Smoking was strongly linked to the domains of physical, psychological, and social relationships. Alcohol consumption was negatively associated with all domains except for the overall perception of health, where it showed no significant association. BMI showed a significant positive association with the Social Relationship domain only. Lack of exercise was strongly negatively associated with all QoL domains.

**Table 6:** Impact of sociodemographic factors, diabetic characteristics and adherence status on the QoL of diabetic patients (using multiple linear regression to analyze predictive variables for different domains of QoL). Data are presented as β value and p value. β - standardized regression coefficient. P value <0.05 was considered statistically significant. BMI: body mass index (kg/m2), QoL: quality of life; Ref: reference group.

Variable	Physical domain		Psychological domain		Social relationship domain		Environmental domain		The overall perception of QoL		The overall perception of health	
	β	p value	β	p value	β	p value	β	p value	β	p value	β	p value
Age (ref = > 60 year age)	-0.12	0.02	-0.12	0.02	-0.06	0.24	-0.14	0.01	-0.16	0.03	-0.03	0.63
Gender (ref = male)	-0.02	0.66	-0.03	0.48	-0.03	0.46	-0.04	0.40	-0.02	0.76	0.01	0.81
Education (ref = illiterate)	0.07	0.15	0.08	0.12	0.12	0.02	0.07	0.19	0.03	0.70	0.11	0.11
Current Occupation (ref = housework and unemployed)	0.08	0.07	0.15	0.00	0.16	0.00	0.14	0.00	0.05	0.44	-0.10	0.12
Marital status (ref = separated, widow, divorced)	0.00	0.92	-0.03	0.50	0.01	0.78	-0.02	0.56	-0.01	0.90	0.00	0.95
Smoker (ref = non-smoker)	0.10	0.02	0.11	0.01	0.11	0.01	0.07	0.10	0.09	0.15	0.07	0.24
Alcoholic (ref = non-alcoholic)	-0.11	0.00	-0.11	0.01	-0.18	0.00	-0.13	0.00	-0.12	0.01	-0.10	0.06
BMI	0.00	0.94	-0.03	0.50	0.10	0.01	0.03	0.48	0.04	0.52	-0.02	0.71
Not doing exercise (ref = doing exercise)	0.20	0.00	0.23	0.00	0.12	0.01	0.20	0.00	0.22	0.00	0.17	0.00
Family h/o diabetes (ref = no family h/o of diabetes)	0.09	0.01	0.08	0.04	0.11	0.00	0.11	0.00	0.10	0.07	0.06	0.22
Type of diabetes (ref = type 1)	0.10	0.02	0.09	0.04	0.12	0.00	0.11	0.01	0.08	0.15	0.14	0.01
Duration of diabetes (ref => 5 yrs)	0.04	0.35	-0.04	0.29	-0.03	0.44	-0.01	0.79	-0.03	0.58	0.08	0.16
Presence of diabetes complication (ref = no any complication)	-0.06	0.18	0.00	0.91	-0.04	0.38	-0.05	0.28	-0.01	0.85	-0.01	0.91
Presence of comorbidity (ref = no comorbidity)	0.18	0.00	0.17	0.00	0.11	0.01	0.14	0.00	0.03	0.57	0.17	0.00
Medication adherence (ref = nonadherence)	0.43	< 0.001	0.36	< 0.001	0.47	< 0.001	0.44	0.00	0.40	< 0.001	0.41	< 0.001

Those who have a positive family history of diabetes were significantly associated with better scores in all domains except the overall perception of health. Type of diabetes was significantly positively associated with all domains except for the overall perception of health. The duration of diabetes did not show significant associations in any domain. The presence of diabetes complications and comorbidities were not significantly associated with most domains. The physical, psychological, and social relationship domains displayed a significant positive correlation with the existence of comorbidities. However, medication adherence was strongly positively associated with all QoL domains, indicating that treatment adherence had a substantial impact on patients' lives.

## Discussion

The WHOQoL-BREF scale was adopted in this study to assess the QoL among diabetic patients for numerous reasons. It is not specifically for diabetes, but it is generally applicable and has been proven effective in Indian languages. Furthermore, with a Cronbach's alpha score of 0.78, the WHOQoL-BREF exhibits good internal reliability, confirming its validity as an assessment tool. Because of its usefulness, many researchers favor it [15].

In the study, mean scores across physical, psychological, and social domains were around 43-44, with the environment domain slightly lower at around 42. In contrast, the overall perception of QoL and health scored around 42. Some studies in India found a similar range of mean scores in the QoL of diabetes patients. Mishra et al. found that diabetes significantly impaired psychological and physical QoL domains, with the mean score of overall perception of QoL and overall perception of health is 63.97 ± 16.51 and 68.16 ± 14.69 respectively [16]. Reasons for variation in study results will be discussed in the subsequent text.

Nonadherence to antidiabetic medications and QoL

In the present study, participants with poor adherence to antidiabetic medication had lower mean scores across all domains of QoL, with notable increases in scores observed from nonadherence to high adherence. Physical domain, mean scores increased significantly from nonadherence (22.59 ± 6.99) to high adherence (82.26 ± 8.55). Similarly, the mean score of psychological, social relationship, and environmental domains also increased from nonadherence to high adherence. The overall perception of QoL mean score increased significantly from nonadherence (20.27 ± 15.3) to high adherence (82.14 ± 19.07) and increased the overall perception of health mean score from nonadherence (17.57 ± 13.55) to high adherence (83.93 ± 12.2). Mishra et al. and Majeed et al. found that medication adherence independently predicts QoL, with adherent patients reporting significantly higher overall QoL perceptions compared to nonadherent patients [16-17]. Many studies have shown a link between medication adherence status and QoL, indicating improved patient outcomes with adherence-enhancing interventions [18-19]. However, some studies have not supported this conclusion [20-21]. Inconsistent findings may arise from variations in study populations, settings, and measurement tools for QoL and adherence. Disease-specific instruments for QoL assessment may be more effective for changes compared to generic measures.

Enhanced medication adherence can significantly enhance QoL by maximizing medication benefits, ultimately leading to reduced symptoms and improved disease control [21]. Adherence can positively impact the QoL by encouraging patients to actively manage their disease through treatment, contributing to their overall well-being continually weighing the benefits and drawbacks of adherence, even in the face of initial difficulties, and modifying their behavior accordingly.

Patients may stop taking medication when they feel better or worse, thinking that changes in QoL indicate treatment effectiveness or ineffectiveness, potentially affecting adherence [22,23]. The correlation between adherence and QoL may be circular. Nonadherence can reduce QoL, leading to further nonadherence. Research has also found that factors like perceived competence and autonomous self-regulation influence both QoL and adherence [23,24].

Other factors associated with QoL

In the present study, older age, higher education, current occupation, BMI and smoking showed significant association with QoL scores. Lack of exercise was strongly negatively associated with all QoL domains. Furthermore, participants with type 2 diabetes, a positive family history of diabetes, and participant have comorbidity showed significant association with QoL scores.

In a comprehensive review conducted by Aarthy et al. and compared with other relevant studies, it was found that several risk factors are associated with a diminished quality of life in type 2 diabetes patients [15]. These risk factors include female gender, a history of stroke, longer duration of diabetes, poor glycemic control, complications related to neuropathy and nephropathy, as well as the use of a combination of oral hypoglycemic agents and insulin. Manjunath et al.​​ observed poor QoL among females, widowed, having a lower socioeconomic status [25].

Tamornpark et al. reported that individuals with diabetes have moderate to low QoL in the mental and physical domains, but good WHOQoL-BREF scores in the environmental and social relationship domains [26]. Factors associated with good QoL include younger age, higher income, nuclear family, regular exercise, disease knowledge, absence of complications, and government medical support. Similarly, Mishra et al. highlighted associations between higher QoL scores and factors such as postgraduate education, higher income, closer proximity to healthcare facilities, longer duration of type 2 diabetes, and specific medication regimens [16]. Chantzaras et al.​ show lower QoL associated with female gender, lower socioeconomic status, lower education, unemployment, higher BMI, sedentary lifestyle, higher HbA1c (glycosylated hemoglobin) levels, type 2 diabetes, and a greater number of comorbidities [27].

Regularly evaluating the QoL in diabetic patients is essential for clinical management. Improving knowledge about diabetes and its medications positively affects adherence. Medication adherence can be enhanced through patient counseling, disease education, and simplifying dosing regimens, leading to significant health and economic benefits [17]. The QoL of diabetic patients was found to be significantly influenced by many factors, including education level, age, work, familial history of diabetes, drinking alcohol and smoking, exercise habits, and medication adherence. Out of all the QoL domains, medication adherence demonstrated the strongest positive association.

Limitations

The study's cross-sectional design limits establishing causal relationships and only provides the prevalence of nonadherence and QoL. Efforts to ensure sample representativeness may not fully generalize to other populations. Data collection relied on participant recall, introducing potential recall bias. Subjective measures used to assess QoL and medication adherence may affect the reliability of results. Additionally, FBS (fasting blood sugar), PP2BS (post-prandial blood sugar), and HbA1C were not measured to assess blood sugar levels, precluding an examination of the correlation between adherence and its effect on diabetic patients.

## Conclusions

This study has demonstrated a high rate of nonadherence to antidiabetic medications. Patients with poor adherence had significantly lower QoL scores. Improving medication adherence through counseling and simplifying dosing regimens can lead to significant health and economic benefits. The analysis highlights the critical role of sociodemographic factors and disease characteristics in shaping the QoL for diabetic patients. Interventions focusing on improving medication adherence and promoting healthier lifestyle choices, such as regular exercise and smoking cessation, may significantly enhance the overall well-being of diabetic individuals. These findings emphasize the need for personalized care strategies that consider these factors to improve patient outcomes.

## Appendices

Appendix A

Antidiabetic Medication Adherence Scale

The antidiabetic medication adherence scale was used to check the adherence status of study participants.

**Table 7:** Antidiabetic medication adherence scale. The internal consistency of the scale was high, as indicated by a Cronbach’s alpha value of 0.91 for all 10 items. The total score of the scale falls within the range of 0 to 40, with nonadherence categorized as 31-40, mild adherence as 21-30, moderate adherence as 11-20, and high adherence as 0-10.

Sr No.	Questions	Never (0)	Rarely (1)	Occasionally (2)	Frequently (3)	Very frequently (4)
1.	How often do you forget to take your anti-diabetic medications?	0	1	2	3	4
2.	How often do you skip taking your antidiabetic medication in case you feel better?	0	1	2	3	4
3.	How often do you omit taking your antidiabetic medication when you're feeling ill?	0	1	2	3	4
4.	How often do you forget to take your antidiabetic medication when you are advised to take it more than once a day then previous one?	0	1	2	3	4
5.	How often do you delay getting your antidiabetic medication refilled?	0	1	2	3	4
6.	How often do you ignore to refill your antidiabetic medication before they run out?	0	1	2	3	4
7.	How often do you decide to take less of your antidiabetic medication dosage than your doctor has prescribed?	0	1	2	3	4
8.	How often do you stop taking your daily antidiabetic medication without telling your doctor?	0	1	2	3	4
9.	How often do you forget to bring along your antidiabetic medication when you travel away from home?	0	1	2	3	4
10.	How often do you change the dose of your antidiabetic medication according to the intake of sweets?	0	1	2	3	4

Appendix B

WHO Quality of Life (QoL)-BREF Questionnaires

Please read the question, assess your feelings, for the last two weeks, and circle the number on the scale for each question that gives the best answer for you.

**Table 8:** WHO Quality of Life (QoL)-BREF questionnaires. WHOQoL-BREF, a 26-item assessment tool developed by the World Health Organization, assesses the QoL across four domains: physical health, psychological, social relationships, and environment. Most of the items utilize a 1 to 5 scale, with higher ratings indicating better QoL. We reversed the negatively phrased items 3, 4, and 26 before conducting additional analysis. A high QoL is indicated by a high score, while a low QoL is indicated by a low scale.

Sr. No.	Question	Very poor	Poor	Neither poor nor good	Good	Very good
1	How would you rate your quality of life?	1	2	3	4	5
Sr. No.	Question	Very dissatisfied	Fairly dissatisfied	Neither satisfied nor dissatisfied	Satisfied	Very satisfied
2	How satisfied are you with your health?	1	2	3	4	5
Sr. No.	Questions	Not at all	A small amount	A moderate amount	A great deal	An extreme amount
3	To what extent do you feel that physical pain prevents you from doing what you need to do?	5	4	3	2	1
4	How much do you need any medical treatment to function in your daily life?	5	4	3	2	1
5	How much do you enjoy life?	1	2	3	4	5
6	To what extent do you feel your life to be meaningful?	1	2	3	4	5
Sr. No.	Questions	Not at all	Slightly	Moderately	Very	Extremely
7	How well are you able to concentrate?	1	2	3	4	5
8	How safe do you feel in your daily life?	1	2	3	4	5
9	How healthy is your physical environment?	1	2	3	4	5
Sr. No.	Questions	Not at all	Slightly	Somewhat	To a great extent	Completely
10	Do you have enough energy for everyday life?	1	2	3	4	5
11	Are you able to accept your bodily appearance?	1	2	3	4	5
12	Have you enough money to meet your needs?	1	2	3	4	5
13	How available to you is the information you need in your daily life?	1	2	3	4	5
14	To what extent do you have the opportunity for leisure activities?	1	2	3	4	5
Sr. No.	Question	Not at all	Slightly	Moderately	Very	Extremely
15	How well are you able to get around physically?	1	2	3	4	5
Sr. No.	Questions	Very dissatisfied	Fairly dissatisfied	Neither satisfied nor dissatisfied	Satisfied	Very satisfied
16	How satisfied are you with your sleep?	1	2	3	4	5
17	How satisfied are you with your ability to perform your daily living activities?	1	2	3	4	5
18	How satisfied are you with your capacity for work	1	2	3	4	5
19	How satisfied are you with yourself?	1	2	3	4	5
20	How satisfied are you with your personal relationships?	1	2	3	4	5
21	How satisfied are you with your sex life?	1	2	3	4	5
22	How satisfied are you with the support you get from your friends?	1	2	3	4	5
23	How satisfied are you with the conditions of your living place?	1	2	3	4	5
24	How satisfied are you with your access to health services?	1	2	3	4	5
25	How satisfied are you with your transport?	1	2	3	4	5
Sr. No.	Question	Never	Infrequently	Sometimes	Frequently	Always
26	How often do you have negative feelings such as blue mood, despair, anxiety, or depression?	5	4	3	2	1
